# Supplementary material for: Longitudinal plasma neurofilament light chain and patient-reported outcomes as complementary markers of vincristine-associated peripheral neuropathy in adults with lymphoma: a cohort study
Source: J Neurol. 2026 Sep 13;273(10):594. doi: 10.1007/s00415-026-14096-2 (PMC13572337; doi:10.1007/s00415-026-14096-2)
Supplement: Supplementary file 1 [file 415_2026_14096_MOESM1_ESM.docx]

**Supplemental figure and table legends**

**Figure S1. Primary NfL model displayed on the log10 scale.**

Model-estimated plasma NfL and 95% confidence intervals from the primary mixed-effects model shown directly on the log10 scale. The model included timepoint as a fixed effect and a participant-level random intercept. Points show model estimates and the shaded band shows the 95% confidence interval. P values are model-based estimated marginal mean contrast p values comparing each follow-up timepoint with T1/Pre-C1. Compared with T1/Pre-C1, log10 NfL was higher at T2/Pre-C2 (p = 4.16e-06) T3/Pre-C4 (p = 6.41e-10) and T4/Pre-C6 (p = 3.39e-08).

**Figure S2. Plasma NfL trajectories by chemotherapy regimen.**

Model-estimated plasma NfL geometric means and 95% confidence intervals by chemotherapy regimen, back-transformed to pg/mL from an exploratory mixed-effects model including timepoint, regimen, and timepoint-by-regimen interaction terms. Points show back-transformed model estimates, and vertical error bars show 95% confidence intervals.

Statistical testing used model-based fixed-effect and interaction terms from the exploratory regimen model. The main effect for EPOCH compared with CHOP was not significant (p = 0.779). Timepoint-by-regimen interaction terms were not significant at T2/Pre-C2 (p = 0.132) T3/Pre-C4 (p = 0.929) or T4/Pre-C6 (p = 0.356) compared with T1/Pre-C1. CHOP, cyclophosphamide, doxorubicin, vincristine, and prednisone; EPOCH, etoposide, prednisone, vincristine, cyclophosphamide, and doxorubicin; NfL, neurofilament light chain.

**Figure S3. Exploratory vincristine dose-modification analyses.**

(A) Change in log10 plasma NfL from baseline to T4/Pre-C6 stratified by whether participants had any vincristine dose modification by T4/Pre-C6. (B) Change in raw CIPN20 total score from baseline to T4/Pre-C6 stratified by any vincristine dose modification (including discontinuation) by T4/Pre-C6. (C) Change in raw CIPN20 total score from baseline to T4/Pre-C6 stratified by any vincristine dose modification (including discontinuation) by T4/Pre-C6.

Boxes show the interquartile range, horizontal lines show the median, whiskers show the observed minimum and maximum, and points show individual participants. Mann-Whitney U tests.

(A) all-participant log10 NfL analysis included n = 11 (3 dose-modified, 8 with no modification) p = 0.1939. (B) all-participant CIPN20 analysis included n = 14 (5 dose-modified, 9 with no modification) p = 0.1381. (C) the outlier-excluded CIPN20 analysis included n = 13 (5 dose-modified, 8 with no modifications) p = 0.2070. The high CIPN20-change outlier was in the no-modification group with delta CIPN20 = +35 from baseline to T4/Pre-C6; this was an IQR-rule outlier within the no-modification group but was retained because it may associate with an observed clinical value. NfL, neurofilament light chain; CIPN20, Chemotherapy-Induced Peripheral Neuropathy questionnaire.

**Table S1. Timepoint-specific associations between plasma NfL and CIPN20 scores**

Spearman correlations between paired log10 plasma NfL and CIPN20 total score are shown separately for T1/Pre-C1,T2/Pre-C2,T3/Pre-C4, and T4/Pre-C6. These analyses were exploratory and underpowered, and were used to contextualize the pooled visit-level association shown in Figure 5A. NfL, neurofilament light chain; CIPN20, Chemotherapy-Induced Peripheral Neuropathy questionnaire.

**Table S2. Subject-level vincristine dose-modification and treatment-period clinical context**

Rows show subject-level visit data for vincristine dose modification, CIPN20 total and sensory scores, BPI-SF, CTCAE, NfL, and the added baseline cancer stage and HbA1c/eA1c context. HbA1c/eA1c combines measured HbA1c with glucose-derived estimated HbA1c when HbA1c was unavailable; glucose-derived eA1c values were used descriptively only. ND: not documented; CIPN20, Chemotherapy-Induced Peripheral Neuropathy 20-item questionnaire; BPI-SF, Brief Pain Inventory-Short Form; CTCAE, Common Terminology Criteria for Adverse Events; NfL, neurofilament light chain; HbA1c, hemoglobin A1c; eA1c, estimated HbA1c.

**Table S3. Exploratory T5/Post patient-reported outcomes and dose-modification context**

Long-term patient-reported outcomes are shown for participants with available T5/Post data 24-42 months after completion of vincristine-containing chemotherapy. Treatment-period CIPN20, T5/Post CIPN20, T5/Post BPI-SF, T4/Pre-C6 CTCAE grade, available treatment-period plasma NfL values, and vincristine dose-modification history are presented descriptively. NfL, neurofilament light chain; CIPN20, Chemotherapy-Induced Peripheral Neuropathy questionnaire; CTCAE, Common Terminology Criteria for Adverse Events; BPI-SF, Brief Pain Inventory-Short Form.

**Figure S1. Primary NfL model displayed on the log10 scale**

**Figure S2. Plasma NfL trajectories by chemotherapy regimen**
**Figure S3. Exploratory vincristine dose-modification analyses.**

**Table S1.** Timepoint-specific association between log10 NfL and CIPN20 scores

| **Timepoint** | **N** | **Spearman ρ** | **p value** |
| --- | --- | --- | --- |
| T1/Pre-C1 | 16 | -0.358 | 0.174 |
| T2/Pre-C2 | 7 | 0.891 | 0.007 |
| T3/Pre-C4 | 17 | 0.399 | 0.112 |
| T4/Pre-C6 | 11 | -0.119 | 0.728 |

**Table S2.** Subject-level vincristine dose-modification, CIPN20 sensory, and treatment-period biomarker context

|  | **Code** | | **Treat-ment** | | **Stage** | | **HbA1c/eA1c (%)** | | **HbA1c/eA1c source** | | **HbA1c/eA1c category** | | **Timepoint** | | **Vincristine dose modification** | | **Dose-modification reason** | | **CIPN**  **20**  **total** | | **CIPN**  **20 sensory** | | **BPI-SF** | | **CTCAE** | | **NfL (pg/mL)** | |
| --- | --- | --- | --- | --- | --- | --- | --- | --- | --- | --- | --- | --- | --- | --- | --- | --- | --- | --- | --- | --- | --- | --- | --- | --- | --- | --- | --- | --- |
| P01 | | CHOP | | IV | | 5.6 | | Measured HbA1c | | Normal | | T1/Pre-C1 | | No modification | | Not applicable | | 19 | | 9 | | 1.5 | | 0 | | 69.9 | |  |
| P02 | | CHOP | | IV | | 6.9 | | Glucose-derived eA1c | | Diabetes range | | T1/Pre-C1 | | No modification | | Not applicable | | 21 | | 10 | | 1 | | 0 | | 187.9 | |  |
| P03 | | CHOP | | IV | | 5.1 | | Measured HbA1c | | Normal | | T2/Pre-C2 | | No modification | | Not applicable | | 25 | | 10 | | 2.5 | | 0 | | 101.6 | |  |
| P03 | | CHOP | | IV | | 5.1 | | Measured HbA1c | | Normal | | T3/Pre-C4 | | No modification | | Not applicable | | 25 | | 11 | | 4.75 | | 0 | | 96.3 | |  |
| P03 | | CHOP | | IV | | 5.1 | | Measured HbA1c | | Normal | | T4/Pre-C6 | | No modification | | Not applicable | | 31 | | 14 | | 5.25 | | 0 | | Missing | |  |
| P04 | | EPOCH | | IV | | 5.4 | | Measured HbA1c | | Normal | | T1/Pre-C1 | | No modification | | Not applicable | | 25 | | 10 | | 0 | | 1 | | 12.9 | |  |
| P04 | | EPOCH | | IV | | 5.4 | | Measured HbA1c | | Normal | | T3/Pre-C4 | | Removed | | CIPN | | 32 | | 14 | | 0 | | 2 | | 175.5 | |  |
| P04 | | EPOCH | | IV | | 5.4 | | Measured HbA1c | | Normal | | T4/Pre-C6 | | Removed | | CIPN | | 35 | | 13 | | 0 | | 1 | | 75 | |  |
| P05 | | CHOP | | IV | | 7.0 | | Measured HbA1c | | Diabetes range | | T1/Pre-C1 | | No modification | | Not applicable | | 19 | | 9 | | 0 | | 0 | | 17.9 | |  |
| P05 | | CHOP | | IV | | 7.0 | | Measured HbA1c | | Diabetes range | | T3/Pre-C4 | | No modification | | Not applicable | | 27 | | 9 | | 1.75 | | 0 | | 70.3 | |  |
| P06 | | EPOCH | | II | | 4.7 | | Glucose-derived eA1c | | Normal | | T1/Pre-C1 | | No modification | | Not applicable | | 21 | | 9 | | 0 | | 0 | | 63.1 | |  |
| P06 | | EPOCH | | II | | 4.7 | | Glucose-derived eA1c | | Normal | | T3/Pre-C4 | | No modification | | Not applicable | | 19 | | 9 | | 0 | | 0 | | Missing | |  |
| P06 | | EPOCH | | II | | 4.7 | | Glucose-derived eA1c | | Normal | | T4/Pre-C6 | | No modification | | Not applicable | | 25 | | 10 | | 0 | | 0 | | 94.4 | |  |
| P07 | | CHOP | | III | | 6.0 | | Measured HbA1c | | Prediabetes | | T1/Pre-C1 | | No modification | | Not applicable | | 19 | | 9 | | 0 | | 0 | | 23.7 | |  |
| P07 | | CHOP | | III | | 6.0 | | Measured HbA1c | | Prediabetes | | T3/Pre-C4 | | No modification | | Not applicable | | 20 | | 9 | | 2 | | 0 | | 114.3 | |  |
| P07 | | CHOP | | III | | 6.0 | | Measured HbA1c | | Prediabetes | | T4/Pre-C6 | | No modification | | Not applicable | | 25 | | 10 | | 0.75 | | 1 | | 172.1 | |  |
| P08 | | EPOCH | | ND | | 5.4 | | Measured HbA1c | | Normal | | T1/Pre-C1 | | No modification | | Not applicable | | 25 | | 10 | | Missing | | 0 | | Missing | |  |
| P09 | | EPOCH | | ND | | 4.2 | | Measured HbA1c | | Normal | | T1/Pre-C1 | | No modification | | Not applicable | | 20 | | 10 | | 0.25 | | 0 | | 72.4 | |  |
| P09 | | EPOCH | | ND | | 4.2 | | Measured HbA1c | | Normal | | T3/Pre-C4 | | No modification | | Not applicable | | 21 | | 10 | | 0.5 | | 1 | | Missing | |  |
| P09 | | EPOCH | | ND | | 4.2 | | Measured HbA1c | | Normal | | T4/Pre-C6 | | 50% reduction | | constipation | | 20 | | 10 | | 0.25 | | 1 | | 189.9 | |  |
| P10 | | CHOP | | IV | | 7.3 | | Measured HbA1c | | Diabetes range | | T2/Pre-C2 | | No modification | | Not applicable | | 21 | | 10 | | 0 | | 0 | | 80.1 | |  |
| P10 | | CHOP | | IV | | 7.3 | | Measured HbA1c | | Diabetes range | | T3/Pre-C4 | | No modification | | Not applicable | | 25 | | 11 | | 0 | | 0 | | 80 | |  |
| P10 | | CHOP | | IV | | 7.3 | | Measured HbA1c | | Diabetes range | | T4/Pre-C6 | | No modification | | Not applicable | | 27 | | 12 | | 0 | | 0 | | 80.8 | |  |
| P11 | | CHOP | | IV | | 6.8 | | Measured HbA1c | | Diabetes range | | T1/Pre-C1 | | No modification | | Not applicable | | 20 | | 9 | | 0 | | Missing | | 31.4 | |  |
| P11 | | CHOP | | IV | | 6.8 | | Measured HbA1c | | Diabetes range | | T3/Pre-C4 | | No modification | | Not applicable | | 21 | | 9 | | 0 | | 0 | | 232.2 | |  |
| P12 | | EPOCH | | ND | | 6.0 | | Measured HbA1c | | Prediabetes | | T1/Pre-C1 | | No modification | | Not applicable | | 22 | | 11 | | 0 | | 0 | | 58.9 | |  |
| P13 | | CHOP | | ND | | 6.5 | | Glucose-derived eA1c | | Diabetes range | | T1/Pre-C1 | | No modification | | Not applicable | | 21 | | 9 | | 2.5 | | 0 | | 16.1 | |  |
| P13 | | CHOP | | ND | | 6.5 | | Glucose-derived eA1c | | Diabetes range | | T3/Pre-C4 | | No modification | | Not applicable | | 26 | | 15 | | 0 | | 1 | | 99.9 | |  |
| P13 | | CHOP | | ND | | 6.5 | | Glucose-derived eA1c | | Diabetes range | | T4/Pre-C6 | | No modification | | Not applicable | | 31 | | 14 | | 0 | | 1 | | 63.2 | |  |
| P14 | | CHOP | | IV | | 6.0 | | Measured HbA1c | | Prediabetes | | T2/Pre-C2 | | 25% reduction | | CIPN | | 21 | | 10 | | 0.5 | | 2 | | 74.3 | |  |
| P14 | | CHOP | | IV | | 6.0 | | Measured HbA1c | | Prediabetes | | T3/Pre-C4 | | 25% reduction | | CIPN | | 23 | | 11 | | 4 | | 1 | | Missing | |  |
| P14 | | CHOP | | IV | | 6.0 | | Measured HbA1c | | Prediabetes | | T4/Pre-C6 | | 50% reduction | | CIPN | | 25 | | 12 | | 4.5 | | 2 | | Missing | |  |
| P15 | | CHOP | | IV | | 4.7 | | Glucose-derived eA1c | | Normal | | T2/Pre-C2 | | No modification | | Not applicable | | 19 | | 9 | | 0 | | 0 | | 68.7 | |  |
| P15 | | CHOP | | IV | | 4.7 | | Glucose-derived eA1c | | Normal | | T3/Pre-C4 | | No modification | | Not applicable | | 18 | | 9 | | 0 | | 0 | | 62.6 | |  |
| P15 | | CHOP | | IV | | 4.7 | | Glucose-derived eA1c | | Normal | | T4/Pre-C6 | | No modification | | Not applicable | | 19 | | 9 | | 0 | | 0 | | 57.4 | |  |
| P16 | | CHOP | | IIIA | | 6.2 | | Measured HbA1c | | Prediabetes | | T1/Pre-C1 | | No modification | | Not applicable | | 20 | | 9 | | 0 | | Missing | | 19.7 | |  |
| P16 | | CHOP | | IIIA | | 6.2 | | Measured HbA1c | | Prediabetes | | T3/Pre-C4 | | 50% reduction | | Multifactorial | | 24 | | 9 | | 1.75 | | 1 | | 74.8 | |  |
| P16 | | CHOP | | IIIA | | 6.2 | | Measured HbA1c | | Prediabetes | | T4/Pre-C6 | | 50% reduction | | Multifactorial | | 19 | | 9 | | 4 | | 0 | | 84.3 | |  |
| P17 | | EPOCH | | IV | | 4.3 | | Measured HbA1c | | Normal | | T1/Pre-C1 | | No modification | | Not applicable | | 20 | | 9 | | 0 | | 0 | | 19.2 | |  |
| P17 | | EPOCH | | IV | | 4.3 | | Measured HbA1c | | Normal | | T3/Pre-C4 | | No modification | | Not applicable | | 20 | | 9 | | 0 | | 0 | | 58 | |  |
| P17 | | EPOCH | | IV | | 4.3 | | Measured HbA1c | | Normal | | T4/Pre-C6 | | No modification | | Not applicable | | 21 | | 9 | | 0 | | 0 | | 53.1 | |  |
| P18 | | EPOCH | | IV | | 4.5 | | Measured HbA1c | | Normal | | T2/Pre-C2 | | No modification | | Not applicable | | 19 | | 9 | | 0 | | 0 | | 36.2 | |  |
| P18 | | EPOCH | | IV | | 4.5 | | Measured HbA1c | | Normal | | T3/Pre-C4 | | No modification | | Not applicable | | 35 | | 20 | | 7.75 | | 1 | | 63.9 | |  |
| P18 | | EPOCH | | IV | | 4.5 | | Measured HbA1c | | Normal | | T4/Pre-C6 | | No modification | | Not applicable | | 54 | | 24 | | 4 | | 0 | | 74.4 | |  |
| P19 | | CHOP | | IV | | 6.3 | | Glucose-derived eA1c | | Prediabetes | | T2/Pre-C2 | | No modification | | Not applicable | | 20 | | 9 | | Missing | | 0 | | 57.7 | |  |
| P19 | | CHOP | | IV | | 6.3 | | Glucose-derived eA1c | | Prediabetes | | T3/Pre-C4 | | No modification | | Not applicable | | 21 | | 9 | | 0 | | 0 | | Missing | |  |
| P19 | | CHOP | | IV | | 6.3 | | Glucose-derived eA1c | | Prediabetes | | T4/Pre-C6 | | No modification | | Not applicable | | 24 | | 9 | | 0 | | 0 | | 84.6 | |  |
| P20 | | EPOCH | | ND | | 5.5 | | Glucose-derived eA1c | | Normal | | T1/Pre-C1 | | No modification | | Not applicable | | 22 | | 9 | | 0 | | 0 | | 12.1 | |  |
| P20 | | EPOCH | | ND | | 5.5 | | Glucose-derived eA1c | | Normal | | T3/Pre-C4 | | Removed | | Ileus | | 18 | | 9 | | 0 | | 0 | | 32.1 | |  |
| P20 | | EPOCH | | ND | | 5.5 | | Glucose-derived eA1c | | Normal | | T4/Pre-C6 | | Removed | | Ileus | | 20 | | 9 | | 0 | | 0 | | Missing | |  |
| P21 | | CHOP | | IV | | 8.5 | | Measured HbA1c | | Diabetes range | | T2/Pre-C2 | | No modification | | Not applicable | | 22 | | 9 | | 0 | | 0 | | 142.2 | |  |
| P21 | | CHOP | | IV | | 8.5 | | Measured HbA1c | | Diabetes range | | T3/Pre-C4 | | No modification | | Not applicable | | 21 | | 9 | | 0 | | 1 | | 67.4 | |  |
| P22 | | CHOP | | ND | | 4.7 | | Measured HbA1c | | Normal | | T2/Pre-C2 | | No modification | | Not applicable | | 22 | | 11 | | 0 | | 0 | | Missing | |  |
| P22 | | CHOP | | ND | | 4.7 | | Measured HbA1c | | Normal | | T3/Pre-C4 | | No modification | | Not applicable | | 20 | | 9 | | 0 | | 0 | | 37 | |  |
| P22 | | CHOP | | ND | | 4.7 | | Measured HbA1c | | Normal | | T4/Pre-C6 | | No modification | | Not applicable | | Missing | | Missing | | Missing | | 0 | | 34 | |  |
| P23 | | EPOCH | | IIA | | 5.4 | | Measured HbA1c | | Normal | | T1/Pre-C1 | | No modification | | Not applicable | | 21 | | 9 | | 0 | | 0 | | 6.8 | |  |
| P23 | | EPOCH | | IIA | | 5.4 | | Measured HbA1c | | Normal | | T3/Pre-C4 | | No modification | | Not applicable | | 26 | | 13 | | 0 | | 0 | | 65.7 | |  |
| P24 | | CHOP | | III-IV | | 5.4 | | Measured HbA1c | | Normal | | T1/Pre-C1 | | 25% reduction | | Not specified | | 18 | | 9 | | Missing | | 0 | | 71.5 | |  |
| P24 | | CHOP | | III-IV | | 5.4 | | Measured HbA1c | | Normal | | T3/Pre-C4 | | 25% reduction | | Not specified | | 26 | | 10 | | 2.25 | | 0 | | 212.8 | |  |
| P25 | | CHOP | | ND | | 5.0 | | Measured HbA1c | | Normal | | T1/Pre-C1 | | No modification | | Not applicable | | 21 | | 9 | | 3.25 | | 0 | | 25.9 | |  |
| P25 | | CHOP | | ND | | 5.0 | | Measured HbA1c | | Normal | | T3/Pre-C4 | | No modification | | Not applicable | | 25 | | 9 | | 5 | | 0 | | 208.6 | |  |

**Table S3.** Exploratory Post/T5 patient-reported outcomes and vincristine dose-modification context

| **Code** | **Treat-ment** | **Months since comple-tion** | **T4/Pre-C6 CIPN20 total** | **T5/Post CIPN20 total** | **T5/Post CIPN20 sensory** | **T5/Post BPI-SF** | **T4/Pre-C6 CTCAE** | **Dose modifica-tion** | **% reduction/ removal** | **Timing/ reason** | **NfL T1/ Pre-C1** | **NfL T2/ Pre-C2** | **NfL T3/ Pre-C4** | **NfL T4/ Pre-C6** |
| --- | --- | --- | --- | --- | --- | --- | --- | --- | --- | --- | --- | --- | --- | --- |
| P06 | EPOCH | 28 | 25 | 20 | 9 | 0.25 | 0 | No | No modification |  | 63.1 |  |  | 94.4 |
| P09 | EPOCH | 42 | 20 | 28 | 16 | 0 | 1 | Yes | 50% reduction | C6; constipation | 72.4 |  |  | 189.9 |
| P13 | CHOP | 38 | 31 | 25 | 13 | 3.5 | 1 | No | No modification |  | 16.1 |  | 99.9 | 63.2 |
| P14 | CHOP | 24 | 25 | 36 | 21 | 4.25 | 2 | Yes | 25% reduction, 50% reduction | C2/C5; CIPN |  | 74.3 |  |  |
| P16 | CHOP | 39 | 19 | 21 | 10 | 1.5 | 0 | Yes | 50% reduction | C4; multifactorial | 19.7 |  | 74.8 | 84.3 |
| P18 | EPOCH | 38 | 54 | 22 | 9 | 2.5 | 0 | No | No modification |  |  | 36.2 | 63.9 | 74.4 |
